# Nigeria media framing of coronavirus pandemic and audience response

**DOI:** 10.34172/hpp.2020.32

**Published:** 2020-07-12

**Authors:** Ekwutosi Sanita Nwakpu, Valentine Okwudilichukwu Ezema, Jude Nwakpoke Ogbodo

**Affiliations:** ^1^Department of Mass Communication, Alex Ekwueme Federal University, Nigeria; ^2^Department of Mass Communication, Ebonyi State University Abakaliki, Nigeria

**Keywords:** COVID-19, Coronavirus, SARS-CoV-2, Media and coronavirus, Framing pandemic

## Abstract

**Background:** Part of the role of the media is to report any issue affecting the society to the masses. Coronavirus has become an issue of transnational concern. The importance of the media in the coverage of coronavirus disease 2019 (COVID-19) in Nigeria and its implications among Nigerian populace cannot be overestimated. This study evaluates how Nigerian media depict the coronavirus pandemic and how the depictions shape people’s perception and response to the pandemic.

**Methods:** The study employed a quantitative design (newspaper content analysis and questionnaire). The content analysis examines the nature of media coverage of coronavirus in Nigeria and China using four major national newspapers (The Sun, The Vanguard, The Guardian and The Punch). The period of study ranged from January 2020 to March 2020. A total of 1070newspaper items on coronavirus outbreak were identified across the four newspapers and content-analysed.

**Results:** The finding shows that the coverage of the pandemic was dominated by straight news reports accounting for 763 or (71.3%) of all analysed items. This was followed by opinions 169(15.8%), features 120 (11.2%) and editorials 18 (1.7%) respectively. The Punch 309 (28.9%)reported the outbreak more frequently than The Sun 266 (24.9%), The Guardian 258 (24.1%), and Vanguard 237 (22.1%). Finding further suggests that the framing pattern adopted by the newspapers helped Nigerians to take precautionary measures.

**Conclusion:** Continuous reportage of COVID-19 has proved effective in creating awareness about safety and preventive measures thereby helping to ‘flatten the curve’ and contain the spread of the virus. However, the newspapers should avoid creating fear/panic in reporting the pandemic.

## Introduction

Scholars have come to the understanding that coronaviruses are zoonotic viruses responsible for mild respiratory tract infections and fatal pneumonia in humans.^1^ Human coronaviruses (HCoV) were first identified in the 1960s in the noses of patients with common cold.^2^ The Centre for Disease Control and Prevention^3^ identified seven coronaviruses that can affect people as: 229E (alpha coronavirus), NL63 (alpha coronavirus), OC43 (beta coronavirus), HKU1 (beta coronavirus), MERS-CoV (the beta coronavirus that causes Middle East respiratory syndrome), SARS-CoV (the beta coronavirus that causes severe acute respiratory syndrome) and SARS-CoV-2 (the novel coronavirus that causes coronavirus disease 2019 or COVID-19).

The novel coronavirus is a current pandemic of coronavirus family that is currently ravaging the whole world. It is a new strain that has not been previously identified in humans.^4^ It was first identified in Wuhan, capital of Hubei Province of China in December, 2019. It was initially from animal sources; however, (it) has subsequently spread between people.^5^ Early genetic analysis of the outbreak revealed that the virus was similar to, but distinct from, SARS-CoV, but the closest genetic similarity was found in a coronavirus that had been isolated from bats.^6,7^

The symptoms of the novel coronavirus have been variously described to include: fever, cough, shortness of breath, and diarrhea.^3,8,9^ But cases of severe infection can result in pneumonia, kidney failure and death.^4,10^ The novel coronavirus is thought to have been transmitted from animals to humans. However, the genetic epidemiology suggests that from the beginning of December, 2019, when the first cases were retrospectively traced to Wuhan, the spread of infection has been almost entirely driven by human-to-human transmission, not the continued spillover.^11^ This human-to-human transmission of the virus is primarily thought to occur through close contacts with infected persons’ respiratory droplets usually generated by sneezing and coughing.

To keep the public informed, the media just like healthcare officers have been working hard to ensure that COVID-19 is combated. Journalists have also been on the frontline reporting the cases, the death toll and the measures masses should take to avoid contracting the virus. From informing the people about the pandemic, the media also strives to shape public opinion about the spread and the precautionary measures that can help to mitigate it (flatten the curve).

Extant literature reveals that some scientific, laboratory and epidemiological studies have been carried out on the outbreak.^12-15^ Given the novel nature of the virus, more related research are expected in the coming months/years. The media play crucial roles in shaping public opinion and perception of issues such as the outbreak of the virus. The way the media frame the pandemic will determine how the public responds to it, and whether to take a precautionary measure or not. Although research focus has been on the outbreak of the pandemic, there is no known empirical study that has focused essentially on the media coverage/framing of the outbreak and how this influences audience perception and response. This is a gap that this study attempts to fill. Also, the fact that no empirical study on the novel coronavirus outbreak from Nigerian background was found in the available literature underscores the need to fill the identified gap. This informs why this study assesses the Nigerian newspapers framing of COVID-19 in selected national newspapers as well as the audience perception and response.

### Mass media and coronavirus

Events of public concern such as health issues definitely attract media attention. This can take the form of print, electronic, internet or social media attention; in most cases, a combination of all. Whichever form it takes, what matters most is the level or impact of media intervention in health crisis, especially disease outbreak. Mass media cannot cure virus but can cure its spread.^16^ This statement explicitly underscores the role of mass media in health reporting, especially in curbing spread of infectious disease outbreak.

Scholars have argued that the mass media have the potential to influence health-related behaviors and perceptions.^17^ In Nigeria, the mass media are used independently or complementarily in health promotion activities to achieve positive lifestyle changes.^18^ Therefore, the role of mass media in health promotion and intervention goes beyond just creating awareness on a particular health issue or disease outbreak; it also entails placing emphasis on the angle or direction of reportage of such issue or outbreak. These media angles of presenting health issues take the form of media framing to influence public perception and induce attitudinal response, leading to positive behavioral changes.

However, research^19^ shows that what always raises dust regarding media roles in health issues is the degree of success or failure recorded by mass media in a particular health outbreak or health challenge compared to the risks. Stressing on this, Tabbaa^20^ asserts that good communication through the media is pertinent when an outbreak is unique in public health with confusion and sense of urgency as the media in no small measure work along with health professional in creating awareness.

Placing priority on novel diseases can be useful because rare and dramatic occurrences are sometimes sentinel events.^21^ Therefore, working with the journalists and the media to help them understand the science and epidemiology, particularly in a fast and moving event, will improve risk communication to the public and reduce inappropriate concerns and panic.^21^

However, previous studies on media reportage of heath crises reveal that even though there exists sufficient scholarly discourse on media reportage of previous disease outbreaks such as Zika virus, influenza, flu, Ebola, and Lassa fever,^16-19,22-24^ no empirical study on media framing of the current coronavirus outbreak was found. The need to fill this lacuna in the existing literature of media reportage of health crises necessitated this present study, and it is coming at a time when the COVID-19 outbreak is on the rapid increase all over the world.

## Materials and Methods

This study adopted a quantitative approach. Content analysis was used to analyze how Nigeria newspaper framed/depicted coronavirus. While questionnaire was employed to further determine how the frame patterns shape audience responses to the outbreak.

### Content analysis 

Content analysis is ‘‘a research technique for the objective, systematic, and quantitative description of the manifest content of communication.’’^25^ It is a ‘‘technique for making inferences by objectively and systematically identifying specified characteristics of messages.’’^26^ The use of content analysis enables us to evaluate the dominant news frames used in reporting COVID-19 in Nigeria. By being systematic, content analysis shows consistency and suppresses bias. This is to ensure replicability of the approach when another researcher applies this approach on the same subject matter.

The newspapers (*The Sun* , *The Vanguard, The Guardian* and *The Punch* ) were selected because they feature high readership, and they are influential in setting the tone for reporting coronavirus pandemic in Nigeria. They are all Nigerian newspapers even though they also featured COVID-19 news about China prominently. By having high circulation rate, these newspapers are highly likely to include broader coverage of the pandemic. Moreover, the newspapers present views from both popular and elite Nigerian publics and they offer a fairly relative representation of different political, geographical and ethnic divides in the country.

The study sample was drawn using the continuous weeks format. Giving that the study period was only three months (1^st^ January to 31^st^ March 2020), the use of continuous week allows us to sample all available COVID-19 related stories published within the period. The continuous week format means that the coverage of the pandemic was examined for all seven days in each week to avoid skipping an important date in the coverage. This yielded a total of 1070 items across the four newspapers. This also served as our sample size. Coding sheet (accompanied by a coding guide) was developed to assess the presence or absence of the frames, with particular attention to news stories, editorials, features, opinions and pictures as units of analysis. To ensure reliability of the coding instrument, Holsti’s^27^ inter-coder reliability formula was adopted to obtain 80% agreement between two independent coders who coded 32 editions (8 each) of the selected newspapers for the pre-test.

### Questionnaire

This instrument was also used in the study to measure the audience response to the coverage of the pandemic. Using a purposive sampling technique, the questionnaires were administered to respondents via six research assistants across the six geopolitical zones in Nigeria. Nigeria has 36 states that make up these six zones. One state was selected from each zone. States covered include Ebonyi (Southeast); Lagos (Southwest); Akwa Ibom (South-south); Jos (Northcentral); Borno (Northeast); and Kano (Northwest). Although we purposively targeted 200 respondents, only 189 were able to complete all the questions and thus, found usable and analyzable. Participants were predominantly within the age range of 21 and 45. The respondents comprised of 118 (62.4%) of males and 71 (37.6%) of females. For reliability purpose, the questionnaire was face-validated by two Professors of Mass Communication before embarking on data collection.

### Data presentation/analysis

For the analysis of data collected using coding sheet, a total of 1070 media messages on coronavirus outbreak were identified across the selected national newspapers and analyzed using SPSS version 20. The newspapers mostly reported the outbreak as news story which has 763 (71.3%) reports, followed by opinions 169 (15.8%), features 120 (11.2%) and editorials 18 (1.7%) having the least reports.

Table 1 above was used to determine the differences in how the selected newspapers reported the novel coronavirus outbreak in China and Nigeria as well as other affected countries. Result from this table reveals that the newspapers reported the outbreak frequently within the study period, with a total of 1070 reports. However, *Punch* 309 (28.9%) reported the outbreak more frequently than *The Sun* 266 (24.9%), *The Guardian* 258 (24.1%) and*Vanguard* 237 (22.1%). Result from Table 1 also indicates the geographical location of coronavirus stories as found in the selected newspapers. The newspapers played their proximity role by frequently reporting the outbreak in Nigeria 489 (45.7%) more than that of the global 325 (30.4%) and China 256 (23.9%) which has the least report, though the outbreak started there before it rapidly spread to other countries and Nigeria.

**Figure 1:**
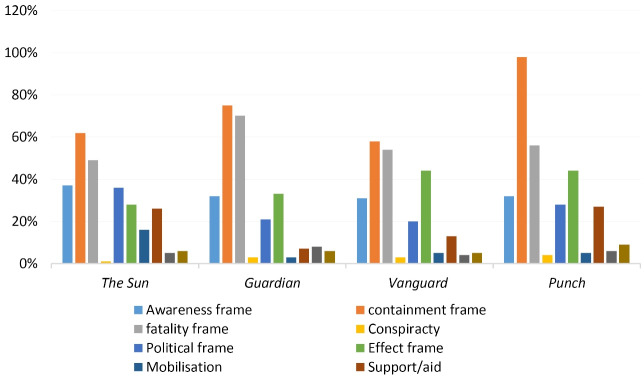
Story frames.

Table 2 and Figure 1 show the patterns of frames adopted by the newspapers in reporting the coronavirus outbreak, and which among the frames is more dominant than others in the reports. The results from the tables indicate that out of ten frames of reports adopted by the selected newspapers in reporting the coronavirus outbreak, containment frame was the most dominant in the reports, as 293 out of 1070 reports on the outbreak were basically on containment efforts, which accounted for 27.4% of the total reports. However,*Punch* 98 (31.7%) presented containment reports more than *The Guardian* 75 (29.1%), *The Sun* 62 (23.3%) and*Vanguard* 58 (24.5%). The second dominant frame adopted by the newspapers was fatality frame which was adopted in 229 reports, accounting for (21.4%) of the total reports. This frame was mostly adopted by *The Guardian* 70 (27.1%), followed by*Punch* 56 (18.1%),*Vanguard* 54 (22.8%) and *The Sun* 49 (18.4%). Other frames of reports adopted by the newspapers include: effect frame 44 (14.2%), awareness frame 32 (10.4%), political influence frame 28 (9.1%), support/aid frame 27 (8.7%), stigmatization/boycott frame 9 (2.9%), misinformation frame 6 (1.9%), mobilization frame 5 (1.6%) and conspiracy frame 4 (1.3%) in *Punch* ; effect frame 44 (18.6%), awareness frame 31(13.1%), political influence frame 20 (8.4%), support/aid frame 13 (5.5%), mobilization frame 5 (2.1%), stigmatization/boycott frame 5 (2.1%), misinformation 4 (1.7%) and conspiracy frame 3. (1.3%) in *Vanguard* ; effect frame 33 (12.8%), awareness frame 32 (12.4%), political influence frame 21(8.1%), misinformation frame 8 (3.1%), support/aid frame 7 (2.7%), stigmatization/boycott frame 6 (2.3%), mobilization frame 3 (1.2%) and conspiracy frame 3 (1.2%) in *The Guardian* ; awareness frame 37 (13.9%), political influence frame 36 (13.5%), effect frame 28 (10.5%), support/aid frame 26 (9.8%), mobilization frame 16 (6.0%), stigmatization/boycott frame 6 (2.3%), misinformation frame 5 (1.9%) and conspiracy frame 1 (0.4%) in *The Sun* .

Tables 3 and 4 were used to ascertain audience perception and response to the framed reports on coronavirus outbreak in the selected newspapers. Out of the 200 distributed questionnaires, 189 were returned and analyzed. In order to ascertain audience perception of the framed reports on the outbreak, the respondents were asked to rate the themes or frames they consider most frequent or dominant in the newspapers they have read as regards the outbreak. Forty-four respondents each, rated containment and fatality frames as the most dominant in the coronavirus reports they have read, accounting for 23.3% each. These were followed by effect frame 26 (13.8%), political influence frame 19 (10.1%), awareness frame 18 (9.5%), stigmatization frame 14 (7.4%), support/aid frame 8 (4.2%), misinformation frame 7 (3.7%), mobilization frame 6 (3.2%) and conspiracy frame 3 (1.6%) as the least rated. This rating depicts a significant relationship between what the media (newspapers) reported or made salient and what the audience perceived from the reports. Also, to determine how they responded to the framed reports in the newspapers, the respondents were asked whether what they read on the newspapers concerning the coronavirus outbreak influenced their action and attitude as regards taking precautionary measures. 172 respondents accounting for 91.0% of the total number of respondents answered “Yes” while only 17 respondents (9.0%) answered “No”.

## Discussion

The main aim of this study is to examine the Nigerian newspapers framing of novel coronavirus outbreak (which originated from China and has subsequently spread to other countries, including Nigeria) with a view to ascertaining how the framing influenced audience perception and response. The frequency and focus of reports were first used to determine how the Nigerian newspapers reported the distant coronavirus outbreak in China and that of Nigeria as well as other affected countries. Findings from the study reveal that the newspapers reported the outbreak in Nigeria (45.7%) more frequently than the similar outbreak in other countries (30.4%) and China (23.9%) where the outbreak started. This suggests that Nigerian newspapers played their social responsibility and proximity roles in this health crisis by frequently reporting the events surrounding the outbreak in their immediate environment than the distant outbreak in China and other countries of the world, thus, recognizing the nearness, importance and relevance of such events to their target audience (Nigerians).

The researchers also identified, in the course of their coding, the patterns of frames adopted by the newspapers in reporting the outbreak. Ten frames of reports regarding the events surrounding the coronavirus outbreak were identified across the selected newspapers. They include: awareness, containment, fatality, conspiracy, political influence, effect, mobilization, support/aid, misinformation and stigmatization/boycott frames. Findings reveal that the newspapers predominantly adopted containment frame. The dominance of containment frame highly depicts media role in containing infectious disease outbreak through the persistent coverage, monitoring and reportage of efforts made by relevant agencies towards preventing or containing the spread of the virus, thus, corroborating the earlier observation of other scholars^16^ that mass media cannot cure disease but can help to educate the masses on the precautionary measures. The second dominant frame adopted by the newspapers was fatality frame. The dominance of this frame in the reports is, undoubtedly, the result of increased fear and tension arising from the reports of persistent rise in the number of confirmed cases and death toll of coronavirus disease, and the consequences it generated from members of the public – for instance, one of the suspected cases in Nigeria was said to have committed suicide possibly because of the fear of being killed sooner than later by the “deadly” or “killer” virus, which has killed thousands of people worldwide as learnt from the media reports. The *Punch’* s and *Vanguard’* s adoption of effect frame more than other two newspapers suggests their high recognition and portrayal of the effects of the outbreak not just on national, regional or global economy, but also on the health, social, political, cultural and all aspects of human development. The implication of this effect frame is that it adds to the fear, panic and tension already generated by the newspapers through fatality frame. Indeed, research has found that ‘‘widely accessed communication channels can help disseminate useful information, reduce unwarranted fear, and facilitate decision making to reduce exposure and susceptibility’’.^27^ We align our finding with this and further argue even though the reportage has been high across the media, it accentuated public fear. Nonetheless, the fear can be indirectly help in preventing the spread of the pandemic.

Interestingly, *The Sun* adopted awareness and mobilization frames more than other three newspapers, as it did not only alert the public of the novel coronavirus disease outbreak, but also stressed the symptoms, mode of transmission and encouraged people to take precautionary or proactive measures more often than other newspapers that created awareness with relatively low mobilization. *The Sun* also adopted political influence frame more than other newspapers.

The high adoption of this frame by the newspaper suggests its emphasis on government’s activeness - both at the national and international levels - towards containing the coronavirus outbreak. However, *The Guardian* adopted misinformation frame more than other newspapers. This is so given that it vividly debunked misinformation, lies, rumors, myths, etc. regarding the spread, treatment or effect of the pandemic which flooded the social media during the study period. This is a core responsibility of the mainstream media which the newspaper has portrayed through its reports, though not generally enough as compared with the increasingly high level of misinformation/ fake news about coronavirus. The adoption of support/aid frame by the newspapers is also significant as it helped to publicize or appreciate financial and material aids donated by concerned, benevolent, patriotic individuals or corporate bodies towards containing the virus. Other least adopted frames include: stigmatization/boycott frame and conspiracy frame which focused on reports of stigmatization/discrimination of coronavirus victims or relations, or boycott (such as travel restrictions) of affected communities, states or countries, and other activities that disrupted the containment efforts or took advantage of the outbreak for certain benefits respectively.

Then to ascertain how the audience perceived and responded to the framed reports on the outbreak, they were asked to rate the themes (frames) they considered most dominant in the reports. Findings from the study show that majority of the respondents (audience) rated containment and fatality frames as the most frequent or dominant themes in the newspapers. This rating depicts a significant relationship between what the media (newspapers) reported or framed and what the audience perceived from the framed reports, which, no doubt, influenced their behaviors towards containing the spread of already perceived fear-inducing, deadly virus. This finding agrees with that of Adelakun^19^ who found a significant relationship between the preponderant frame (containment frame) and audience rating of such frame in his study of Nigerian newspapers framing of 2014 Ebola Outbreak, though, unlike this present study, his study found totally insignificant relationship among audience rating of other remaining frames identified in the study.

Also, to determine audience response in this study, the respondents were asked whether the newspapers reports influenced their action and attitude towards the disease as regards taking precautionary measures. Majority of them responded positively while only few maintained a negative response, depicting that the media reports on the outbreak actually influenced audience perception and response to a greater extent. This finding adds to the scholarship of media framing and further affirms how framing influences audience perception and response.

Therefore, in connection with its main aim which was to examine how the Nigerian newspapers framed the novel coronavirus outbreak and the influence of these frames on the audience response, this study has spurred further debate in this area of research. It established that the media portrayal of the pandemic improved the awareness creation on the precautionary measures for containing the spread of the virus. Although fear cannot be ruled out in the reportage of this pandemic, it indirectly aided peoples’ response to the virus, making them to take necessary measures to manage the new realities.

### Limitations

The reliance on content analysis and questionnaire (quantitative approach) limit our understanding of the reportage of the pandemic. Employing a mixed methods approach, involving qualitative (for instance, the use of in-depth interview or focus group discussion) and quantitative (content analysis and questionnaire) would have broadened our understanding of audience reaction to the framing of the pandemic. The reason is that in-depth interview offers participants more holistic opportunity to discuss the situation in greater details, and interviewers can ask follow-up questions that the closed nature of questionnaire cannot allow. Although the study is useful in the context it was set, the extent to which it can be generalized outside this setting is not known. Nonetheless, it has expanded scholarship of media framing in an area that only a few empirical literature exists.

### Recommendation for further studies 

Since this study examined the media framing of the pandemic in Nigeria and China, subsequent research can look beyond the Nigerian newspapers and extend the content analysis to Chinese newspapers to offer a more robust understanding of the similarity or dissimilarity in the reportage of the pandemic in the two countries. Chinese audiences can also be surveyed to allow for generalization in a global scale.

### Recommendation for policy making

This study has opened a new conversation for both researchers and policymakers. For policymakers and media establishments, the findings markedly differ from existing studies that tend to blame the media for creating panic in the public through sensational reportage. As our study demonstrates, rather than create fear more frequently, Nigeria newspapers adopted a pattern of coverage that helped Nigerians to take precautionary measures against the pandemic. Media organisations can sustain this tempo in covering further health crises and other related issues to keep the public duly informed. Another practical implication of this finding is that healthcare systems benefit from a responsible media coverage of the pandemic because it means that when more persons take precautionary measures to prevent the spread of the virus, cases cannot overwhelm healthcare professionals as it was the case in the US, UK, Spain and Italy where the number of coronavirus patients overwhelmed the available healthcare facilities and professionals. In a setting like Nigeria where the available healthcare facilities only prioritise the wellbeing of the elite class, taking the mediatised precautionary measures really helped to manage the onset of the pandemic in the country.

This study further recommends that:

- The Nigerian newspapers should tilt more of their reports on the outbreak towards positive direction of allaying fear/tension rather than heightening it while trying to contain the spread of the virus; this can be achieved by reporting more of news analysis/features, opinions and editorials on the outbreak.
- The newspapers should go beyond merely creating awareness to mobilizing the people to be more proactive in containing the spread of the virus.

## Conclusion

Based on the above findings, this study concludes that Nigerian newspapers performed their social responsibility role effectively by frequently reporting events surrounding the outbreak, especially containment efforts and awareness creation on the outbreak. The newspapers also recognized the place of proximity in news selection by reporting more frequently the outbreak in Nigeria than that of distant outbreak of similar disease in foreign countries.

However, the newspapers generated high tension, fears and panic on the Nigerian public through frequent adoption of fatality frame in their reports. This situation, if left to continue, could even cause more deaths than the virus itself, thereby worsening the negative impacts of the pandemic on the audience. Indeed, a study by Basch et al^27^ found that wide access to communication channels can help disseminate relevant information, reduce unnecessary fear, and facilitate decision making to reduce exposure and susceptibility. Although the reportage has been high across the media, fatality was foregrounded thereby accentuating public fear. Nonetheless, fear can indirectly help to instill fear in the people thereby preventing the spread of the pandemic.

## Ethical approval

Since eliciting information from the selected media audience was part of the research, the study was deemed human subjects research. Approval was gotten from Department of Mass Communication, Alex Ekwueme Federal University research and ethics Committee Board. The approval number is AE-FUNAI/MAC/EBC/20/0023.

## Competing interests

The authors declare that there is no conflict of interests.

## Funding

We received no funding for this research.

## Authors’ contributions

VOE and ESN sourced the newspaper articles and helped in administering and analysing questionnaires, while JNO helped in putting the content analysis in perspective as well as editing the final draft.

## Acknowledgments

We acknowledge Dr Chike Onwe for providing us with his office space for carrying out this research. We also acknowledge our research assistants: Ezinne, Jacob, Arinze, Yahaya and Tunde for their time in distributing questionnaires.

**Table 1:** Nigeria newspaper reportage of coronavirus and location of story focus

**Newspapers**	**Frequency**	**Percentage**	**Story location**	**Frequency**	**Percentage**
*The Sun*	266	24.9	Nigeria	489	45.7
*Guardian*	258	24.1	China	265	23.9
*Vanguard*	237	22.1	Global (other countries but Nigeria and China)	325	30.4
*Punch*	309	28.9	**Total**	**1070**	**100.0**
**Total**	**1070**	**100.0**			

**Table 2:** Story frames

	**Awareness frame**	**Containment frame**	**Fatality frame**	**Conspiracy frame**	**Political influence frame**	**Effect frame**	**Mobilization frame**	**Support/aids frame**	**Misinformation frame**	**Stigmatization /boycott frame**	**Total**
Newspapers											
*The Sun*	37	62	49	1	36	28	16	26	5	6	266
*Guardian*	32	75	70	3	21	33	3	7	8	6	258
*Vanguard*	31	58	54	3	20	44	5	13	4	5	237
*Punch*	32	98	56	4	28	44	5	27	6	9	309
**Total**	**132**	**293**	**229**	**11**	**105**	**149**	**29**	**73**	**23**	**26**	**1070**

**Table 3:** Audience rating of dominant themes (frames) in the reports

	**Frequency**	**Percent**
Awareness	18	9.5
Containment	44	23.3
Fatality	44	23.3
Conspiracy	3	1.6
Political influence	19	10.1
Effect	26	13.8
Mobilization	6	3.2
Support/aids	8	4.2
Misinformation	7	3.7
Stigmatization	14	7.4
**Total**	**189**	**100.0**

**Table 4:** Influence of newspaper reports of coronavirus on Nigeria audience

	**Frequency**	**Percent**
Yes	172	91.0
No	17	9.0
Total	189	100.0
